# The New York Institute of Stomatology

**Published:** 1903-08

**Authors:** 

**Affiliations:** The New York Institute of Stomatology


### Reports of Society Meetings.

### THE NEW YORK INSTITUTE OF STOMATOLOGY.

A MEETING of the Institute was held at "The Chelsea," No. 222 West Twenty-third Street, New York, on Tuesday evening, April 7, 1903, the President, Dr. J. Morgan Howe, in the chair.

The minutes of the last meeting were read and approved.

Dr. Brockway, the chairman of the committee, presented a memorial of Dr. C. A. Woodward. The memorial was unanimously adopted.

Dr. Geo. B. Terrell read a paper descriptive of a new method of regulating devised by himself.

(For Dr. Terrell's paper, see page 594.)

### DISCUSSION.

Dr. C. O. Kimball spoke of a similarity that existed between this appliance of Dr. Terrell's and the method used by Dr. Baker, of Boston, in that they both made use of the interaction of one jaw upon the other, with this difference, that what Dr. Baker's appliance accomplished by a pulling force, Dr. Terrell's accomplished by pushing. In Dr. Baker's appliance the tendency was constantly to pull the crib off the teeth, and for that reason it was to be cemented on, while pressure of the interaction of the jaws upon the appliance as devised by Dr. Terrell only tended to hold them more firmly in place. The advantage of the latter method was obvious, as it permitted the removal of the appliances during mastication and for the purpose of cleansing. Dr. Kimball presented an appliance of this kind just made for him by Dr. Terrell for a reverse condition,—the protrusion of the upper jaw. It had been found in this case, after the upper jaw had been expanded, that the child could bring her lower jaw forward into correct position, and that when it dropped back it weakened the whole line of her face by the receding chin. The attention of the child's mother, who was anxious to have some teeth extracted, was called to this change in the whole physiognomy when the child bit forward in the proper position, and she was instantly convinced. Judging from the experience of another case, Dr. Kimball was of the opinion that holding the jaws in proper position for three or four weeks' time would so thoroughly fix the habit that it would not be comfortable for them to close in any other position.

Dr. S. A. Hopkins, of Boston, read a paper entitled "Medical Aspects of Dental Lesions."

(For Dr. Hopkins's paper, see page 576.)

DISCUSSION.

Dr. R. H. M. Dawbarn considered the essential features of Dr. Hopkins's paper to be two: First, a very thorough *résumé* of the ways in which troubles about the teeth might have a direct bearing upon disease in general. This was true to a much greater extent than one would realize. On this phase of the subject he could not add anything to the essayist's remarks. The essayist had stated that otitis media was almost always preceded by catarrhal inflammation of the nasal mucous membrane. Dr. Dawbarn was inclined to think that the true cause much more frequently was "adenoids," more properly called lymphoids, of the pharynx. Children who are mouth-breathers are almost always sufferers from this condition, and it was the duty of the dentist to call the patient's, or the patient's parents', attention to it, with the view of having the condition remedied. As high as twenty-five per cent. of all children in such a variable climate as that of the northeast American seaboard were affected with such lymphoid growths.

Regarding the list of microbes found in the human saliva, Dr. Dawbarn remarked that Dr. Nicholas Senn had given about twenty-six of them. Dr. Dawbarn wished to say that Dr. Hopkins had attacked this problem in the right and scientific way, and that in this manner alone do we little by little accomplish anything.

There was one point concerning which Dr. Dawbarn thought we should direct attention and study,—namely, the question why, in regions like the mouth and the rectum, rankly infected continuously by microbes, nevertheless injuries and wounds do not do badly. Given a case of fracture of the lower jaw, and it will in almost all cases be found to be compound, because the gingival mucous membrane is adherent closely to the periosteum, and both are inelastic. Consequently saliva, with its myriad microbes, is all the while bathing the line of fracture. Nevertheless, as a rule, there is good speedy union, and without blood-poisoning. Or, take the case of a fistula in ano, slit open from end to end and necessarily brought in contact with fæces. Such cases are very frequent, and yet the surgeon has little fear of septicæmia in consequence. Why is this? Any fracture elsewhere in the body treated to a continuous bath of saliva would do badly, indeed, without a doubt (unless it is the patient's own saliva), and any wound elsewhere contaminated daily by pouring fæces over it would probably develop erysipelas or some other ugly microbic invasion.

Dr. Dawbarn was of the opinion that the explanation of this contradiction lies in the fact that from birth until death the regions under discussion are vaccinated, so to speak, with ptomaines and toxines, the products of the life activities and death decompositions of the germs normal and native to that region; and because of such vaccinations, the outcome of the numerous slight lesions so frequently occurring, the flesh thereabouts is in some way made better able to combat and overcome any ill results when greater wounds are received in the mouth or rectum. Then too it is a fact worth noting that each healthy individual's saliva is not dangerous to himself, even elsewhere than the mouth, wounds, or fractures, though this secretion would be perilous to another individual. Probably the reason for this is the one already mentioned,—a continual vaccination. We have all seen wounds upon the hands of street urchins sucked and washed clean by their spittle, just as a dog cleanses himself, and with no ill result.

Dr. E. A. Bogue was quite surprised that Dr. Hopkins did not bring other things into his paper. Within the last few weeks there had come under his observation three, perhaps more, cases where the necessity for a better understanding between medical and dental men became very apparent. The last case had presented only a few days since, a lady with an impacted lower molar tooth that somebody had undertaken to remove. In this case the mouth was capable of being opened a little over a quarter of an inch. Dr. Bogue had recommended cold applications, but the lady went away and used hot applications, with the result that within forty-eight hours she was not expected to live. He knew of a young man who had died from the same cause a few months ago.

Dr. C. O. Kimball stated that it had come to his observation, as to others', no doubt, that disease entirely outside the mouth gave rise often to symptoms in the mouth. Dr. Kimball was reminded of a case reported to the society some years ago, a case of herpes zoster, where the symptoms simulated precisely the symptoms of pulp-stone or of pulpitis. This was in line with what Dr. Hopkins had suggested, that we should know more thoroughly the relation of the teeth with the general system. In the case he had just spoken of there had been consultation with some of the best surgeons in New York beforehand. Their judgment had been equally at fault with his. There were so many cases coming up all the time, requiring us to know the relations of the teeth to the general system, that the suggestions of such a paper as Dr. Hopkins's were very valuable.

Regarding the second half of the paper, it seems as if Dr. Hopkins had reached the point that many of us had been striving for years to reach,—viz., proving in a scientific manner, and not merely by actual daily practice, that cleanliness of the teeth affects the general health, which has been a matter of common clinical observation.

The President thought we were greatly indebted to Dr. Hopkins for this presentation of such an interesting subject. Dr. Howe related an experience in his own practice confirmatory of one phase of Dr. Hopkins's paper. The case was that of a young man with a fine set of teeth, who presented himself with a history of pain in a tooth sufficient to keep him awake. He located the pain in a certain lower bicuspid tooth that was perfectly sound with the exception of a small gold filling. Tests of ice and percussion failed to reveal anything but the normal response, and after much persuasion the man was induced to let it alone till the afternoon. He did not appear until the next morning, when he again complained of pain in that tooth, and was so urgent that something be done that the tooth was drilled into far enough to show normal sensitiveness of dentine, when Dr. Howe was confirmed in the opinion that the cause of pain must be looked for elsewhere. He advised consulting his physician. The next morning the patient presented himself with a story of pain in a totally different tooth. He was then told that it was very evident that no tooth at all was at fault, and that he should see his physician for constitutional treatment. The pain eventually subsided without any tooth whatever being operated upon. Dr. Howe cited this case as illustrating the point made in Dr. Hopkins's paper of the necessity of discriminating between diseases of the teeth and diseases of some other parts indirectly affecting the teeth.

Dr. H. L. Wheeler mentioned the case of a young woman who came to him with a swelling in the gum of a lower cuspid tooth. She had been to a dentist several times recently and although he had removed the calcic deposit from her teeth, he had told her that there was nothing the matter with this particular tooth. The tooth itself was perfectly sound and there was no history of an injury and no pockets around the tooth. There was the characteristic odor of pus infection about the patient. Upon opening the tooth a putrescent pulp of long standing was found and a small quantity of very thick pus exuded. In this case the lack of a proper diagnosis endangered the patient's life.

Dr. Leo Green, in this connection, mentioned several cases he had seen this winter where acute intestinal constipation resulted in ulcerative stomatitis, all of which was completely eradicated by proper doses of castor oil.

Dr. Hopkins, in closing, thanked the Institute for its courtesy in discussing the paper. He had purposely omitted mentioning the subject of adenoids in the mouth, as he had previously brought that subject before the society, and he thought it a repetition to mention it now.

Dr. Hopkins thought that testimony given by patients concerning the dental practitioner whom they had last consulted should always be held with a little suspicion. He had never known a patient, in all his practice, to get anything of this kind straight. By a too credulous belief in these stories a great deal of injury might be done to a brother practitioner. A case in point was that of a patient who presented himself with a story of the apparent inability on the part of a brother practitioner to treat a dead tooth. When Dr. Hopkins attempted to open the tooth there was so much resistance on the part of the patient that Dr. Hopkins finally told him that he could readily see why the other dentist had failed, and added that unless he was willing to submit to his method of treatment he must go elsewhere and go quickly. It was quite possible that a man beginning practice might not have the effrontery to talk to a patient in this manner.

Another patient had come from a well-known specialist in Boston suffering from severe neuralgic pains which the specialist thought might come from the teeth. The teeth were strong and good, but were covered with tartar. Dr. Hopkins told the patient that while the removal of the tartar would probably have little effect on her condition, still it was indicated and it should be thoroughly removed. She thereupon went immediately to his friend the specialist and stated that Dr. Hopkins had said that it was merely a matter of tartar, and as soon as that was removed she would be all right.

Upon motion a vote of thanks was extended to Dr. Hopkins.

Adjourned.

FRED. L. BOGUE, M.D., D.D.S.,
*Editor The New York Institute of Stomatology.*

